# Study of the Utility and Impact of Plasma *p*‐tau181 Alzheimer's Biomarkers ‐ SUIT‐ABLE: Patient and Care Partner Outcomes

**DOI:** 10.1002/alz70856_099787

**Published:** 2025-12-24

**Authors:** Abigail Perelman, Megan Zhang, Cameron Coykendall, Kristin Harkins, Jason Karlawish, Kyra O’Brien

**Affiliations:** ^1^ University of Pennsylvania Perelman School of Medicine, Philadelphia, PA, USA; ^2^ University of Pennsylvania, Philadelphia, PA, USA

## Abstract

**Background:**

Early and accurate diagnosis of Alzheimer's disease (AD) affords access to appropriate care, which has the potential to slow symptom progression and encourage future planning. AD diagnostic biomarkers can facilitate early diagnosis. Blood‐based, or plasma, AD biomarkers are rapidly emerging and can enable wider access to AD biomarker testing and therefore early and accurate diagnosis. Phosphorylated‐tau181 can differentiate between AD and non‐AD neuropathological cases, and can predict future cognitive decline in patients with mild cognitive impairment. Plasma AD biomarkers have the potential to significantly impact future AD diagnosis and care; however, before plasma AD biomarker tests can be widely disseminated, the evidence base supporting their clinical use must be built by evaluating their impact in real‐world clinical settings. This study evaluated the impact of plasma *p*‐tau181 testing on patients and their care partners in a memory clinic.

**Method:**

Patients with a syndromic diagnosis of mild cognitive impairment or dementia completed the Quanterix LucentAD plasma *p*‐tau181 assay. Patients and study partners (SPs) completed depression and anxiety scales (PHQ‐9 and PROMIS Anxiety Short Form) prior to learning their test result. The PHQ‐9, PROMIS Anxiety Short Form, Impact of Event Scale – Revised, and comprehension and utility of results surveys were administered at 1‐3 days and 2‐3 months after plasma *p*‐tau181 result disclosure.

**Result:**

Of the 94 patients who completed plasma *p*‐tau181 testing, 81 patients (age 71.0 ± 8.3 years, 55.6% female) and 83 SPs (age 61.0 ± 15.7 years, 55.4% female) completed baseline questionnaires. 73 patients (age 70.8 ± 8.2, 54.8% female) and 69 SPs (age 60.9 ± 14.8, 56.5% female) completed 1‐3 day follow‐up surveys. SPs demonstrated a significant increase in anxiety scores after receiving test results (baseline vs. 1‐3 days post‐disclosure, *p* =  0.003). There was no significant change in depression scores for SPs or in anxiety or depression scores for patients. The majority of patients and SPs found the test results to be informative and useful (patients: 65.1% and 60.3%; SPs: 76.6% and 75.0%).

**Conclusion:**

While plasma AD biomarkers provide useful information for patients and their care partners, adequate supports must be in place to address distress related to the test results.

